# Gap-free genome assembly of Salangid icefish *Neosalanx taihuensis*

**DOI:** 10.1038/s41597-023-02677-z

**Published:** 2023-11-04

**Authors:** Yanfeng Zhou, Xizhao Zhang, Jianbo Jian, Chenhe Wang, Di’an Fang, Shulun Jiang, Long Ren, You Ge, Hongqi Wang, Yang You, Chunhai Chen

**Affiliations:** 1 Key Laboratory of Freshwater Fisheries and Germplasm Resources Utilization, Ministry of Agriculture and Rural Affairs, Freshwater Fisheries Research Center, Chinese Academy of Fishery Sciences, Wuxi, 214081 China; 2 Wuxi Fisheries College, Nanjing Agricultural University, Wuxi, 214081 China; 3 BGI Genomics, BGI-Shenzhen, Shenzhen, 518083 China

**Keywords:** Agricultural genetics, Biodiversity

## Abstract

*Neosalanx taihuensis* is widely distributed in freshwater and brackish water areas in China. Due to its high commercial value, it has been artificially introduced into many lakes and reservoirs, showing strong ecological adaptability. Here, a gap-free chromosome-level reference genome was constructed by combining short reads, PacBio HiFi long reads, Nanopore ultralong reads and Hi-C data. The reference genome of *N. taihuensis* was 397.29 Mb with a contig N50 of 15.61 Mb. The assembled sequences were anchored to 28 chromosomes. Furthermore, 20,024 protein-coding genes and 98.16% of the predicted genes were annotated in publicly available biological databases. This high-quality gap-free assembled genome will provide an essential reference for studying the evolution and ecological adaptability of *N. taihuensis*.

## Background & Summary

*Neosalanx taihuensis*, a member of the Salangidae family of the Osmeriformes, is an economically important aquaculture fish in China with a transparent body and feeds on zooplankton^1–3^. *N. taihuensis* is endemic to fresh and brackish waters widely distributed in China and has been artificially introduced to numerous lakes and reservoirs due to its high commercial value^4^. The natural population is not only distributed in the estuary area from the Yellow and Bohai Seas to the coast of the South China Sea but also in the main streams of the Yangtze River, Huai River and Yellow River and their subsidiary lakes^5,6^. Among these sites, the Yangtze River basin and Taihu Lake are the core habitats of the natural population of *N. taihuensis*^7–9^. The natural *N. taihuensis* population size has declined seriously due to overfishing and habitat destruction^10^. Fortunately, artificial translocation activities for *N. taihuensis* have greatly expanded the spatial-geographic distribution and population diversity of the species^7,11–13^. Translocation activities in waters such as the Erhai Sea, Fuxian Lake, Dianchi Lake and the Three Gorges Reservoir have resulted in the formation of stable populations of *N. taihuensis* in these new habitats^8,11,14,15^. The study of genetic diversity between translocated and natural populations has become an interesting issue for researchers, and a variety of molecular markers, including COI, microsatellites, and Cytb, have been developed^7,9,11,16,17^. The analysis of these markers has shown that the genetic diversity of the translocated population of *N. taihuensis* was higher than that of the natural population and has preliminarily revealed the molecular mechanism of *N. taihuensis* adaptation to the environment^7,9,11,16,17^.

More specifically, translocated *N. taihuensis* also exhibit plasticity in their reproductive biology. In natural habitats such as the Yangtze River basin and Taihu Lake, *N. taihuensis* commonly has two breeding groups, a spring breeding group and an autumn breeding group^18–23^, with the spring breeding group being the main source of population supplementation^21,24^. In contrast, the reproductive pattern of the translocated population of *N. taihuensis* has changed. The reproductive behavior of the translocated *N. taihuensis* population in Erhai shows only one spawning period, i.e., from late autumn to early winter^22^. In contrast, the translocated population of *N. taihuensis* in Dianchi has formed three reproductive groups, namely, the winter group, the autumn group and the spring group^23^. The translocated population has a longer reproductive period than the natural population, showing a more obvious adaptation in reproductive strategy. The current research on the differentiation of reproductive populations of *N. taihuensis* is limited to the description and statistics of epigenetic phenomena, and few studies have investigated the relevant genetic mechanism and molecular evolution.

Until now, molecular biology and genomic research on *N. taihuensis* has been rare due to the lack of a reference genome. The lack of information on the *N. taihuensis* genome greatly limits the study of *N. taihuensis* phylogeny and genetic differentiation. Likewise, it is not possible to explore the adaptation and reproductive strategies of *N. taihuensis* at the genomic level.

In this study, we report a gap-free genome assembly for *N. taihuensis* combining short reads, PacBio HiFi long reads, Nanopore ultralong reads and Hi-C data. The assembled *N. taihuensis* genome was approximately 397.29 Mb with a contig N50 of 15.61 Mb. Gene annotation yielded 20,024 protein-coding genes, and 98.16% of the predicted genes were annotated in publicly available biological databases, including NR, GO, KOG, KEGG, TrEMBL, Interpro and SwissProt. This high-quality, gap-free assembled genome will provide an important resource for studying the reproductive biology and ecological adaptability of *N. taihuensis*.

## Methods

### Ethics declarations

This work was approved by the Bioethical Committee of Freshwater Fisheries Research Center (FFRC) of the Chinese Academy of Fishery Sciences (CAFS) (FEH20200807, 2020/08/07). Sampling was performed in strict accordance with Freshwater Fisheries Research Center Experimental Animal Ethics Guidelines.

### Sample collection

**Fig. 1:**
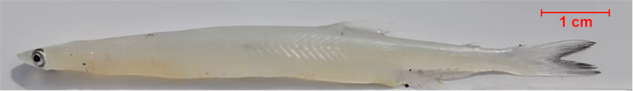
Demonstration image of *N*. *taihuensis*.

Muscle tissue samples were collected from adult *N. taihuensis* for this study (Fig. 1). The collection site was located at Taihu Lake, Huzhou, Zhejiang Province (coordinates: E120°5′0.999996″, N31°0′59.999976″). Sampling was performed in strict accordance with relevant Chinese laws and experimental ethical guidelines. After the muscle tissue samples were collected, they were rapidly frozen in liquid nitrogen and stored at −80 °C until DNA extraction.

DNA and RNA extraction, library construction, sequencing, assembly, and bioinformatics analyses in this study were performed using standard experimental and analytical protocols from BGI Genomics (Shenzhen, China).

### RNA isolate, cDNA library construction and sequencing

For gene structure annotation, RNA was isolated from the muscle tissue samples using the TRIzol Total RNA Isolation Kit (Takara, USA) following the manufacturer’s protocols^25^. Then, the RNA was sheared and reverse transcribed using random primers to obtain cDNA, which was used for library construction. The library quality was determined using a Bioanalyzer 2100. Subsequently, these libraries underwent paired-end sequencing with a read length of 150 bp on the BGISEQ sequencing platform (BGI).

### WGS library construction, sequencing and genome survey

**Fig. 2:**
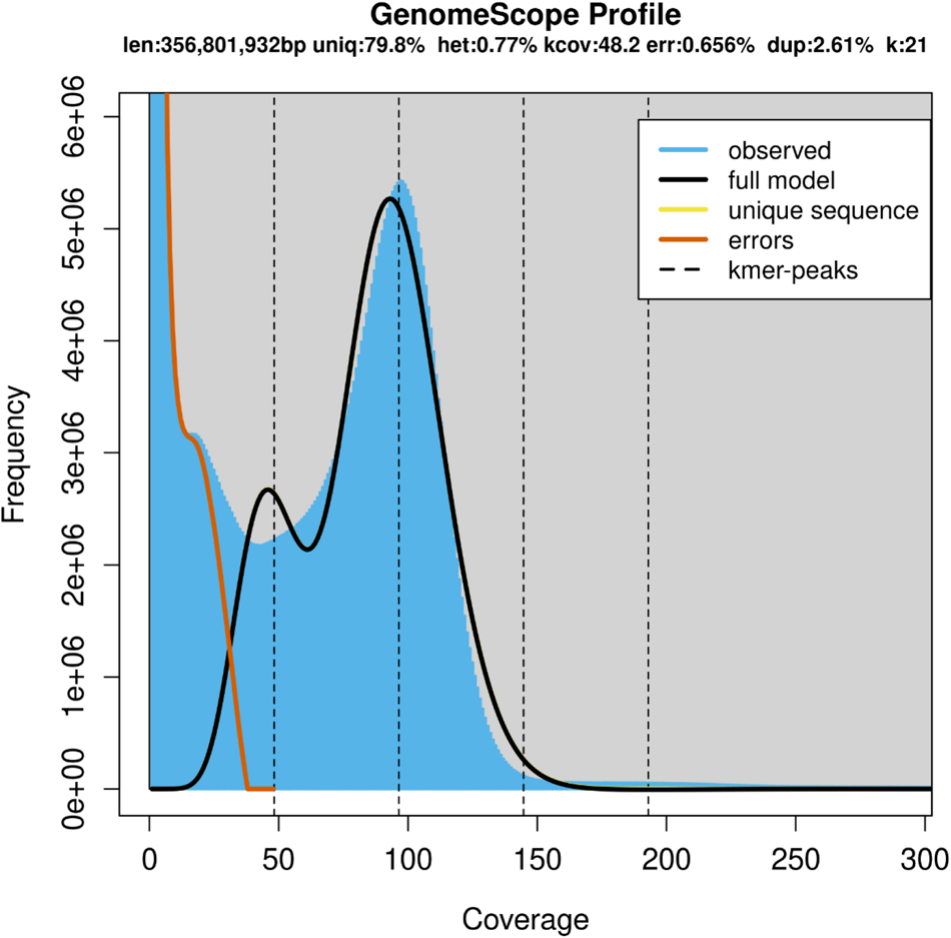
K-mer analysis of *N*. *taihuensis* genome.

Extracted DNA from *N. taihuensis* muscle tissue using hypervariable minisatellite probe (MZ 1.3), along with locus-specific minisatellite probes (g3, MS1, MS43). Fragmented this DNA between 50 and 800 bp using a Covaris E220 ultrasonicator, following manufacturer guidelines, creating a short insert whole-genome shotgun (WGS) library. Built and sequenced a library with fragments between 300 and 400 bp on the MGISEQ platform. Generated 45.69 Gb DNBSEQ data for short inserts, offering insights into the *N. taihuensis* genome (Table 1). Utilized the FastQC (v0.1)^26^ to remove low-quality or adapter-linked reads. From the refined data, determined the K-mer frequency distribution using Jellyfish (v2.2.6)^27^ and analyzed with GenomeScope (v1.0)^28^. Determined the *N. taihuensis* genome to be around 356 Mb with a heterozygosity rate of 0.77% (Fig. 2 and Table 2).

**Table 1:** Sequencing data used for the genome *N. taihuensis* assembly.

Type	Sample	Platform	Bases (Gb)	Reads Count	Max length (bp)	Mean length (bp)	N50 (bp)
CCS	muscle	PacBio Sequel II (Hifi)	25.88	1,735,116	46,637	14,914	15,177
ONT	muscle	Nanopore	15.01	461,934	443,683	32,490	56,103
Hi-C	muscle	DNBSEQ-T7	69.21	461,400,000	150	150	150
WGS	muscle	DNBSEQ-T7	45.65	304,356,646	150	150	150
RNA		DNBSEQ-T7	75.09	500,600,000	150	150	150

**Table 2:** The information of genome survey analysis.

Property	Minimum	Maximum
Heterozygosity	0.77%	0.77%
Genome haploid length	356,502,699	356,801,932
Genome repeat length	71,851,996	71,912,305
Genome unique length	284,650,704	284,889,627
Model Fit	86.84%	1
Read Error Rate	0.66%	0.66%

### PacBio library construction, sequencing and *de novo* assembly

DNA from N. taihuensis muscle tissue was extracted using a QIAGEN Blood & Cell Culture DNA Midi Kit (QIAGEN, Germany). A PacBio library with an insert size of around 20 kb was then prepared using the SMRTbell Express Template Prep Kit 2.0 from PacBio (Pacific Biosciences, USA). It was sequenced on a PacBio Sequel II SMRT cell in CCS mode. After processing with the SMRT Link (v8.0.0)^29^ CCS algorithm with parameters “--minPasses 3 --minPredictedAccuracy 0.99 --minLength 500”, 25.88 Gb HiFi reads were obtained, excluding adaptors and less accurate reads. The reads had an N50 length of 15.17 kb and an average length of 14.91 kb (Table 1). The initial genome *de novo* assembly was done using Hifiasm (v0.15.1)^30^ with standard settings, and any redundant sequences were later purged using the Purge-Haplotigs^31^ program with the parameters “-j 80 -s 80 -a 75”.

### Hi-C library preparation, sequencing and chromosome anchoring

A Hi-C library was created using the Mbo I restriction enzyme^32^. Muscle tissue samples underwent 1% formaldehyde treatment at room temperature for 10–30 minutes to crosslink chromatin-interacting proteins. Post-digestion with Mbo I restriction enzyme (NEB, Ipswich, USA), fragment ends were flattened, repaired, biotin-labeled, and ligated to form loops using T4 DNA ligase (Thermo Scientific, USA). After protein removal and ultrasound disruption of the loops, the Hi-C library was sequenced on an MGISEQ platform. For the chromosome-level assembly, 69.21 Gb of Hi-C sequencing data were produced, leading to the clustering, ordering, and orientation of contigs into 28 pseudochromosomes using Juicer (v1.5)^33^ and 3D-DNA (v180922)^34^ pipelines (Table 1). Scaffolding errors were later reviewed and curated using Juicebox (v1.11.08)^33^.

### Oxford Nanopore Technologies library preparation, sequencing and assembly

**Fig. 3:**
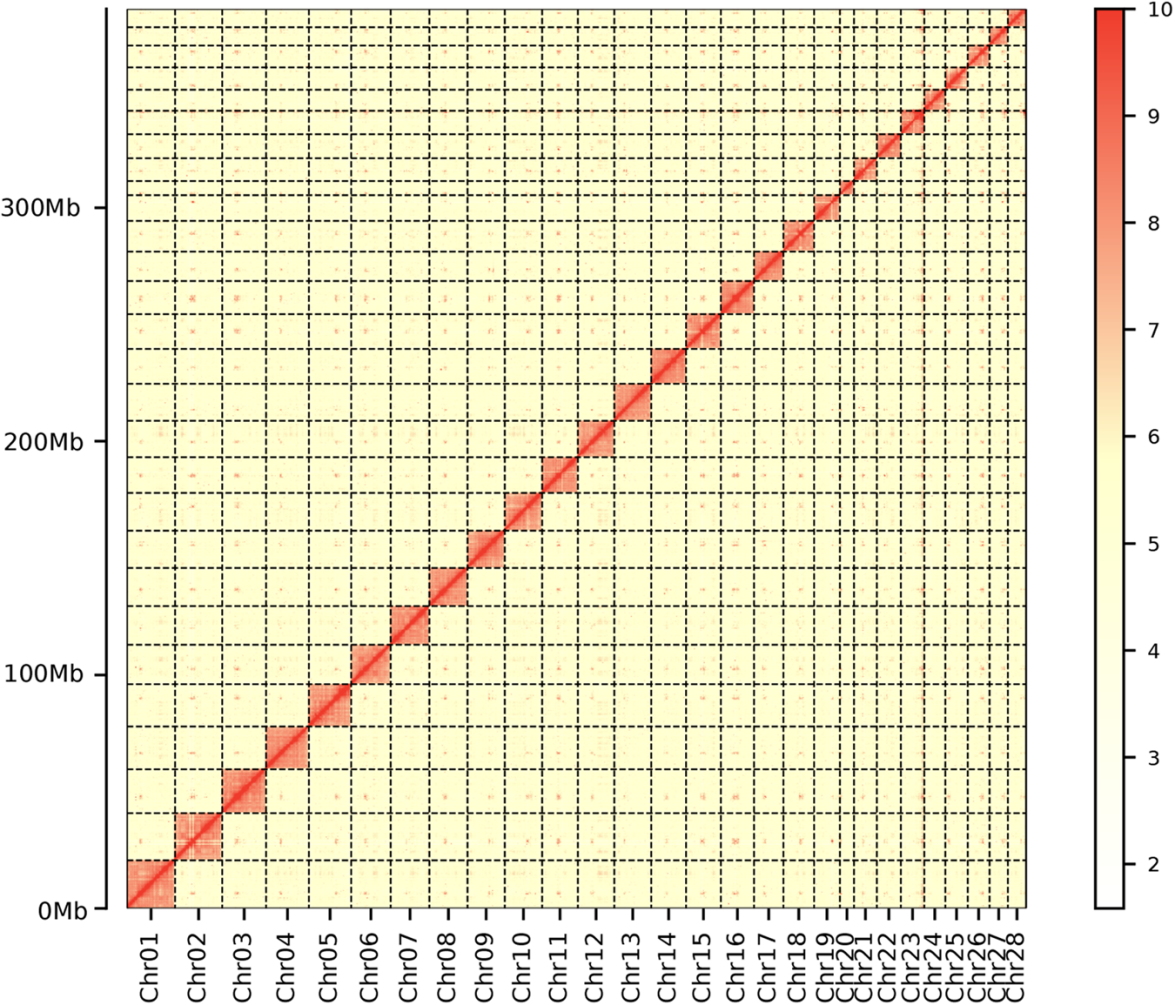
Characteristics of the *N. taihuensis genome*. Hi-C chromatin interaction map of the *N. taihuensis* assembly.

An ultralong library was created using Oxford Nanopore Technologies (ONT). Genomic DNA from *N. taihuensis* muscle tissue was extracted via the CTAB method, focusing on fragments over 5 kb, with SageHLS HMW library system. This DNA was processed using the Ligation sequencing 1D kit (SQK-LSK109) and sequenced on the Promethion platform at the Genome Center of Grandomics (Wuhan, China). ONT ultralong reads were refined, discarding those shorter than 5 kb or with QV below 7. This yielded around 15.01 Gb of ultralong reads with an N50 of 32.49 kb. Errors in these reads were corrected using the Necat pipeline (v 20200119)^35^. The revised ultralong reads then filled gaps in the *N. taihuensi*s assembly using three iterations of LR_Gapcloser (v1.0) with the parameter “--max_distance 1000000 – coverage 0.8 – tolerance 0.2” and one round of TGSgapcloser (v 1.0.1) pipeline with the parameter “--min_idy 0.3”^36,37^. Finally, a gap-free chromosome-level assembly was generated with a genome size of 397.29 Mb and a contig N50 of 15.61 Mb (Table 3). The concluding *N. taihuensis* assembly was generated with PacBio, ONT and HiC data and formed 28 contigs representing 28 chromosomes (Fig. 3, Table 4).

**Table 3:** The statistics of length and number for the *de novo* assembled of *N. taihuensis, P. chinensis and P. hyalocranius* genomes.

Genome	Type	Total length (bp)	Max length (bp)	Number > = 2000bp	N50		N90	
					Length (bp)	Number	Length (bp)	Number
*N. taihuensis*	**scaffold/contig**	397,288,650	20,583,638	137	15,609,449	12	9,007,007	25
*P. chinensis*	**scaffold**	466,693,640	44,188,582	618	5,188,763	23	794,666	110
	**contig**	444,873,684	2,137,849	11,196	103,007	876	8,371	7,942
*P. hyalocranius*	**scaffold**	536,559,363	5,398,389	681	1,163,487	126	397,112	451
	**contig**	414,848,732	366,845	19,755	17,737	5,716	2,822	28,932

**Table 4:** Statistics of chromosomal level assembly of *N. taihuensis* genome.

ID	Length (bp)	N content (%)	GC content (%)	Gap number
Chr01	20,583,638	0	0.46	0
Chr02	20,203,675	0	0.46	0
Chr03	18,817,646	0	0.46	0
Chr04	18,333,975	0	0.46	0
Chr05	18,150,050	0	0.46	0
Chr06	16,877,868	0	0.46	0
Chr07	16,606,454	0	0.46	0
Chr08	16,323,931	0	0.47	0
Chr09	15,970,593	0	0.47	0
Chr10	16,007,973	0	0.47	0
Chr11	15,312,806	0	0.46	0
Chr12	15,609,449	0	0.46	0
Chr13	15,830,736	0	0.47	0
Chr14	14,929,474	0	0.46	0
Chr15	14,897,035	0	0.46	0
Chr16	14,205,173	0	0.46	0
Chr17	12,602,976	0	0.47	0
Chr18	13,135,266	0	0.47	0
Chr19	11,040,416	0	0.47	0
Chr20	6,052,715	0	0.48	0
Chr21	9,778,622	0	0.47	0
Chr22	10,270,060	0	0.47	0
Chr23	10,048,254	0	0.46	0
Chr24	9,007,007	0	0.47	0
Chr25	9,616,609	0	0.47	0
Chr26	9,361,535	0	0.47	0
Chr27	7,825,227	0	0.47	0
Chr28	7,829,588	0	0.48	0
UnChr	12,059,899	0	0.50	0

### Repetitive sequence annotation

**Fig. 4:**
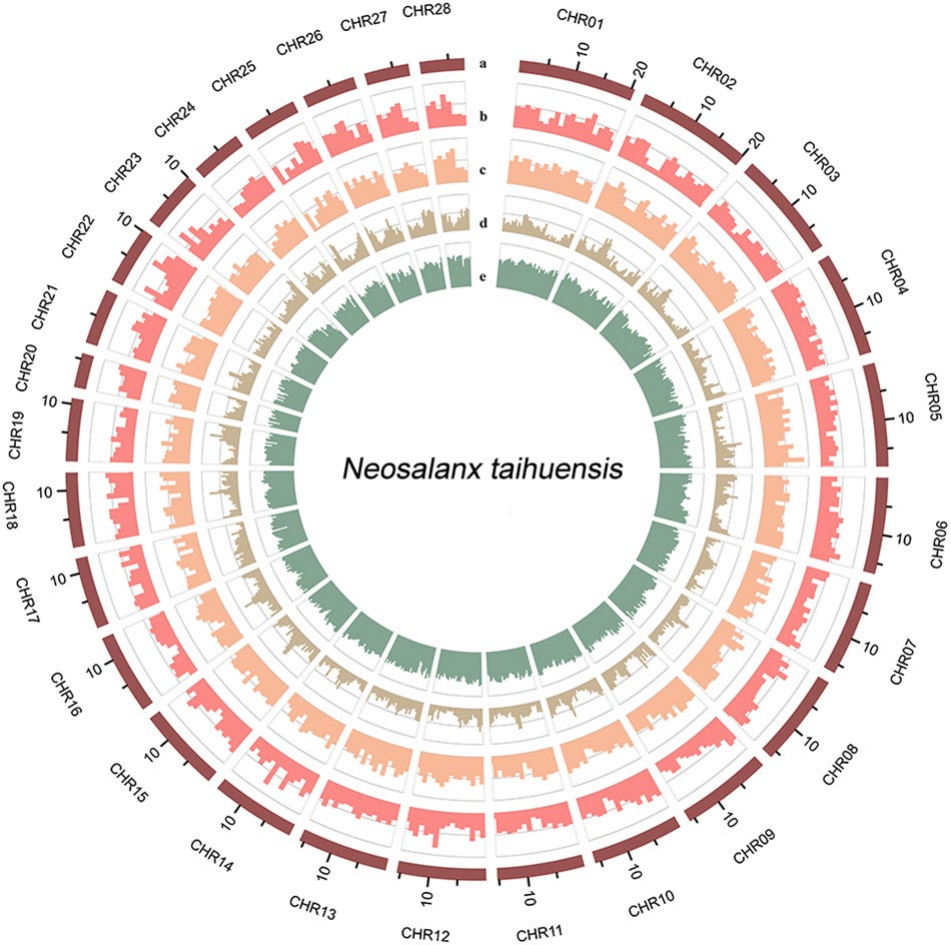
Circos plot of the *N. taihuensis genome*. The rings from inside to outside indicate (**a**) pseudochromosome length of the *N. taihuensis* genome, (**b**) gene frequency, (**c**) gene density, (**d**) TE density, and (**e**) GC density; b-d were drawn in 500-kb sliding windows.

Following genome assembly, repetitive sequences were annotated (Fig. 4). Using RepeatModeler (v1.0.4)^38^ and LTR-FINDER (v1.0.7)^39^ with default parameters, repetitive elements and long terminal repeats were identified. By merging these findings, a *de novo* repeat sequence library was constructed. This library was then used to screen for interspersed repeats and low-complexity sequences via RepeatMasker (v4.0.7)^40^. For homolog-based prediction based on the Repbase database^41^, DNA and protein transposable elements (TEs) were detected by RepeatMasker (v4.0.7) and RepeatProteinMasker (v4.0.7)^40^, respectively. Tandem repeats were identified with Tandem Repeat Finder (v4.10.0)^42^. In total, 149.89 Mb (~37.73%) of repetitive sequences were recognized. For the predominant categories of transposable elements (TEs), long interspersed nuclear elements (LINEs) constituted 11.64% of the *N. taihuensis* genome, DNA hAT transposon (DNA/hAT) elements constituted 7.13%, long terminal repeats Copia (LTRs/Copia) constituted 0.36%, long terminal repeats Gypsy (LTRs/Gypsy) constituted 4.34%, and short interspersed nuclear elements (SINEs) constituted 3.05% (Table 5).

**Table 5:** Statistics of repetitive sequences in the *N. taihuensis* genome.

Type	Repeat size (bp)	Percentage of genome (%)
*Identification method*		
Trf	62,506,186	15.73
Repeatmasker	60,043,004	15.11
Proteinmask	8,276,664	2.08
*De novo*	125,809,332	31.67
Total	149,892,271	37.73
*Biological classification*		
Retro/LTR/Copia	1,444,422	0.36
Retro/LTR/Gypsy	17,245,940	4.34
Retro/LTR/Other	29,305,482	7.38
Retro/SINE	12,115,925	3.05
Retro/LINE	46,234,707	11.64
Retro/Other	50,433	0.01
DNA/EnSpm	11,216,772	2.82
DNA/Harbinger	4,153,138	1.05
DNA/hAT	28,322,177	7.13
DNA/Helitron	10,593,310	2.67
DNA/Mariner	451,292	0.11
DNA/MuDR	513,010	0.13
DNA/P	219,961	0.06
DNA/Other	46,557,873	11.72
Other	14,158,829	3.56
Unknown	1,550,285	0.39
Total	132,072,927	33.24

### Protein-coding gene annotation and functional annotation

To predict protein-coding genes, three strategies, including transcriptome-based annotation, homology-based annotation and *ab initio* prediction, were conducted. For the transcriptome-based annotation, 75.09 Gb RNA-seq data were mapped to the *N. taihuensis* assembly with Hisat2 (v2.1.0)^43^, and then the transcriptome information in BAM alignments was produced. The BAM alignments were further assembled into transcripts using Stringtie (v1.3.5)^44^ and validated by PASA (v2.5.2) (https://github.com/PASApipeline/PASApipeline). Subsequently, coding sequences were identified by TransDecoder (v5.5.0) (https://github.com/TransDecoder/TransDecoder) with default parameters. Assemblies and gene annotation files of four Actinopterygii species (*Danio rerio*, *Oryzias latipes*, *Protosalanx hyalocranius* and *Salmo salar*) were downloaded from a public database (Table 6). According to previous studies^5^, *P. hyalocranius* and *P. chinensis* are actually the same species. Gene annotation files, combined with the RNA-seq BAM alignments and the homolog assemblies, were utilized to conduct homology-based prediction with GeMoMa (v1.8)^45^. Based on the protein homology information, August (v3.2.1)^46^ and SNAP (v2006-07-28) (https://github.com/KorfLab/SNAP) were used to train the predictors. Then, *ab initio* prediction was generated by the August and SNAP programs with the self-training parameters. The EVidenceModeler (EVM) pipeline (v 1.1.1)^47^ was used to integrate all the protein-coding genes predicted by the above three strategies. Finally, the protein-coding genes that were only derived from *ab initio* prediction were filtered out. Overall, 20,400 protein-coding genes were obtained with an average gene length of 8,921 bp and an average CDS length of 1,673 bp. The average exon number per gene was 9, with an average exon length of 177 bp and an average intron length of 858 bp (Table 7).

**Table 6:** The genome information of four actinopterygii species.

Species	Data Accession	Data Source
*Protosalanx hyalocranius*	https://ftp.cngb.org/pub/gigadb/pub/10.5524/100001_101000/100262/	GigaDB
*Protosalanx chinensis*	GCA_010882115.1	NCBI
*Danio rerio*	GCF_000002035.6	NCBI
*Oryzias latipes*	GCF_002234675.1	NCBI
*Salmo salar*	ICSASG_v2	Ensembl-100

**Table 7:** Gene annotation of *N. taihuensis* genome via three methods.

Method	Gene set	Gene number	Average gene length (bp)	Average cds Length (bp)	Average exon num	Average exon Length (bp)	Average intron Length (bp)
***De novo***	Augustus	26,816	5,847	1,277	7	187	784
***De novo***	Snap	56,479	6,708	854	5	163	1,384
**Homolog**	*D. rerio*	21,149	12,762	1,648	9	174	1,313
**Homolog**	*O. latipes*	21,823	12,586	1,649	9	183	1,365
**Homolog**	*P. hyalocranius*	19,588	12,983	1,602	8	195	1,578
**Homolog**	*S. salar*	26,331	12,364	1,662	9	179	1,293
**Transcript**	PASA	58,611	10,326	1,343	7	187	1,455
**Merge**	EVM	24,257	7,515	1,503	9	175	794
**Final set**	—	20,400	8,921	1,673	9	177	858

**Fig. 5:**
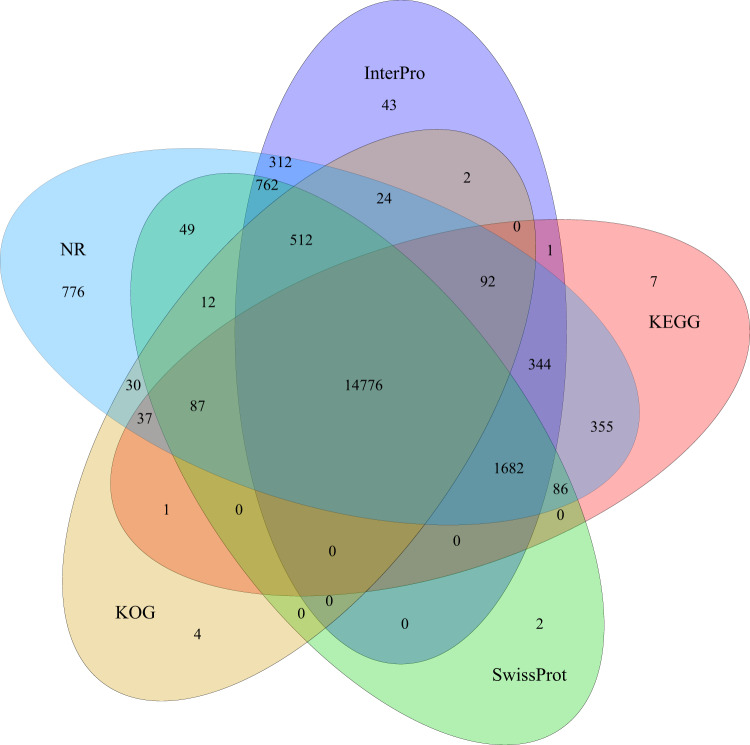
Venn diagram of the number of genes with homology or functional classification by each method. The Venn diagram shows the shared and unique annotations among InterPro, KEGG, KOG, NR and SwissProt.

The final gene models predicted above were then annotated using the NCBI nonredundant (NR) protein database (97.3%) and the Swissprot^48^ (88.08%), KEGG^49^ (85.63%), KOG^50^ (76.36%), TrEMBL^49^ (97.64%), InterPro^51^ (90.93%) and Gene Ontology (GO)^52^ (67.44%) databases. In total, 20,024 (98.16%) gene models were annotated for at least one homologous hit by searching against these public databases (Table 8). Of 20,024 functional proteins, 14,776 (~72.4%) were supported by the data of five databases (InterPro, KEGG, NR, KOG, SwissPort) (Fig. 5).

**Table 8:** Functional annotation statics.

Values	Total	Nr	Swissprot	KEGG	KOG	TrEMBL	Interpro	GO	Overall
Number	20,400	19,936	17,968	17,468	15,577	19,918	18,550	13,758	20,024
Percentage	100%	97.73%	88.08%	85.63%	76.36%	97.64%	90.93%	67.44%	98.16%

## Data Records

All the raw data for the whole genome have been deposited into the National Center for Biotechnology Information (NCBI) SRA database (Accessions for SRR22936158 to SRR22936161) under BioProject accession number PRJNA915819^53^. The Whole Genome Shotgun project has been deposited at GenBank under accession JARGSH000000000^54^.

The files for *N. taihuensis* gene structure annotation, gene functional annotation and repeat annotation have been deposited at Figshare^55^.

## Technical Validation

### Evaluation of the genome assembly

To compare the assembled metrics for *N. taihuensis* and the other Salangidae species, the assembly in this study was to the gap-free chromosome-scale assembly level (Table 3). The contig N50 of our assembly was 15.61 Mb, while that of *P. chinensis*^56^ was 103.01 Kb and that of *P. hyalocranius*^57^ was 17.74 Kb. The contig number for our assembly was 137, while that of *P. chinensis* was 11,196 and that of *P. hyalocranius* was 19,755. These statistics indicated that our assembly had reached a higher contiguous level (Table 3).

The completeness was evaluated using BUSCO^58^ analysis. BUSCO analysis revealed that 91.5% (single-copied gene: 90.0%, duplicated gene: 1.5%) of 3,640 single-copy orthologs (in the actinopterygii_odb10 database) were successfully identified as complete, 1.8% were fragmented and 6.7% were missing in the assembly (BUSCO v5.1.0). The accuracy rate was evaluated by mapping the sequencing data to the assembled genome. The mapping rates were 94.63%, 99.8% and 100% for the DNBSEQ, PacBio data and Nanopore data, respectively.

### Evaluation of the gene annotation

**Fig. 6:**
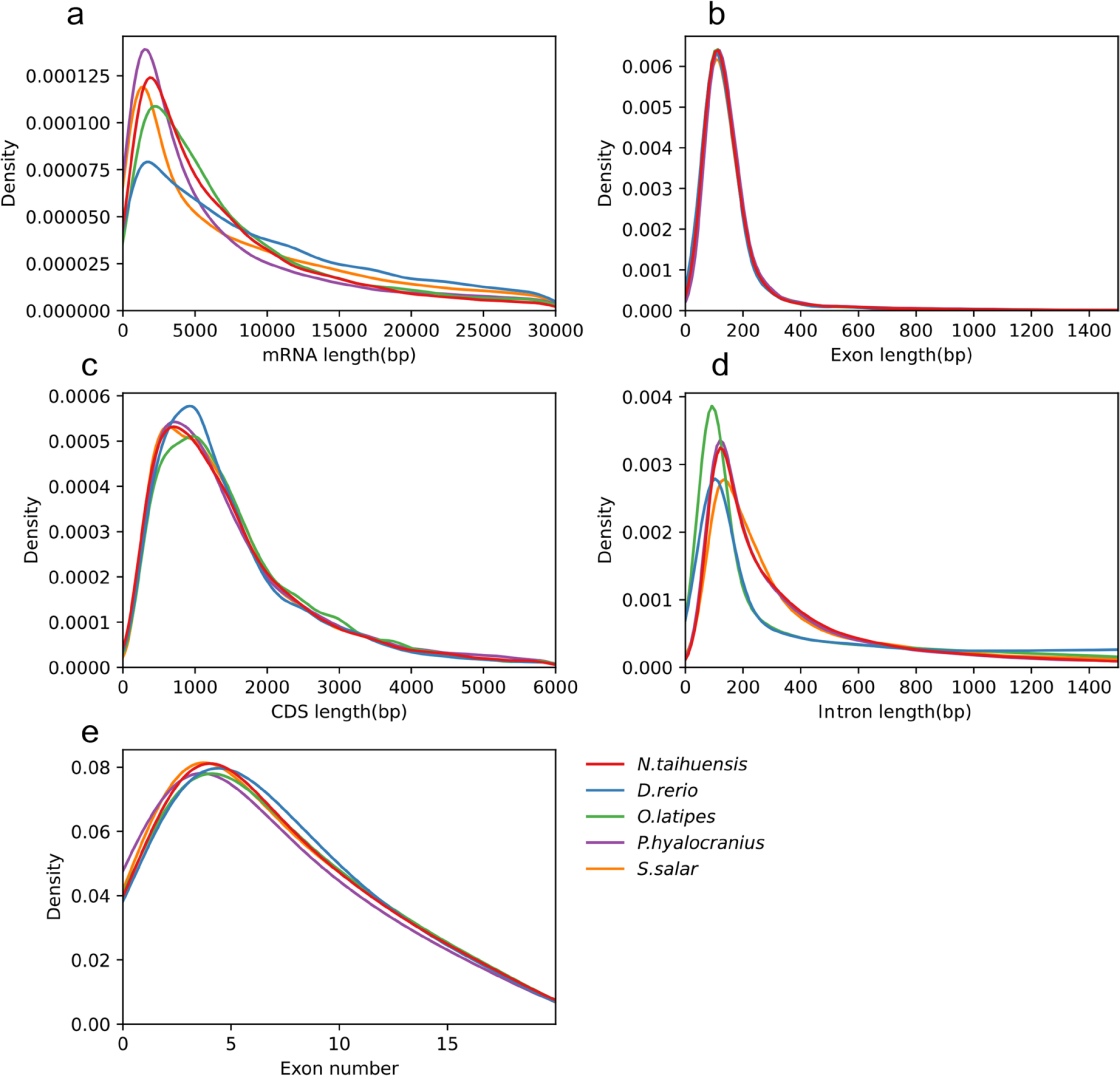
The composition of gene elements in the *N. taihuensis* genome compared to the genomes of other species. (**a**) mRNA length distribution and comparison with other species. (**b**) Exon length distribution and comparison with other species. (**c**) CDS length distribution and comparison with other species. (**d**) Intron length distribution and comparison with other species. (**e**) Exon number distribution and comparison with other species.

The completeness and accuracy of the gene structure annotation were evaluated using three different strategies. First, BUSCO analysis revealed that 90.9% (single-copy gene: 88.5%, duplicated gene: 2.4%) of 3,640 single-copy orthologs (in the actinopterygii_odb10 database) were successfully identified as complete, while 1.6% were fragmented and 7.5% were missing in the assembly (BUSCO v5.1.0) (Table 9). Second, to determine if there was evidence of *de novo* annotation, homolog-based annotation and transcripts, we calculated the CDS overlap content between the final gene sets with the prediction results from the above three different methods. The results showed that more than 99.78% of genes were occupied by these three prediction results with a CDS overlap ratio greater than 80% (Table 10). Moreover, we compared the length distribution of genes, coding sequences (CDS), exons and introns among the *D. rerio*, *O. latipes*, *P. hyalocranius* and *S. salar* genomes and found similar distributions of these parameters (Fig. 6).

**Table 9:** BUSCO Evaluation.

Type	Genome Assembly		Protein-coding gene models	
	Number	Rate (%)	Number	Rate (%)
Complete BUSCOs (C)	3,331	91.5	3,310	90.9
Complete and single-copy BUSCOs (S)	3,276	90	3,223	88.5
Complete and duplicated BUSCOs (D)	55	1.5	87	2.4
Fragmented BUSCOs (F)	66	1.8	57	1.6
Missing BUSCOs (M)	243	6.70%	273	7.50%

**Table 10:** The evidence supporting gene models of the *N. taihuensis* genome.

—	> = 30%	overlap	> = 50%	overlap	> = 80%	overlap
—	Number	Percent (%)	Number	Percent (%)	Number	Percent (%)
C(single)	12	0.06	17	0.08	16	0.08
C(more)	0	0	0	0	0	0
H(single)	5	0.02	9	0.04	69	0.34
H(more)	77	0.38	100	0.49	182	0.89
P(single)	4	0.02	3	0.01	166	0.81
P(more)	1324	6.49	1789	8.77	2936	14.39
HC	161	0.79	236	1.16	646	3.17
PC	607	2.98	542	2.66	487	2.39
PH	2263	11.09	2474	12.13	2902	14.23
PHC	15936	78.12	15209	74.55	12952	63.49
Total	20389	99.95	20379	99.9	20356	99.78

## Data Availability

All software used in this work is in the public domain and the parameters are clearly described in the Methods section. Where no detailed parameters have been mentioned for a type of software, default parameters were used as suggested by the developer.
